# Characterization of the Lytic Phage Flora With a Broad Host Range Against Multidrug-Resistant *Escherichia coli* and Evaluation of Its Efficacy Against *E. coli* Biofilm Formation

**DOI:** 10.3389/fvets.2022.906973

**Published:** 2022-06-13

**Authors:** Liming Jiang, Yaxian Jiang, Wen Liu, Rui Zheng, Chenghua Li

**Affiliations:** ^1^State Key Laboratory for Quality and Safety of Agro-products, Ningbo University, Ningbo, China; ^2^Collaborative Innovation Center for Zhejiang Marine High-Efficiency and Healthy Aquaculture, Ningbo University, Ningbo, China; ^3^Department of Clinical Laboratory, The First People's Hospital of Yunnan Province, Kunming, China; ^4^Department of Clinical Laboratory, The Affiliated Hospital of Kunming University of Science and Technology, Kunming, China; ^5^Department of Rheumatology Immunology, The First People's Hospital of Hefei, Hefei, China; ^6^Laboratory for Marine Fisheries Science and Food Production Processes, Qingdao National Laboratory for Marine Science and Technology, Qingdao, China

**Keywords:** *Escherichia coli*, phage, host range, biofilm, antibiotic

## Abstract

*Escherichia coli* is a gram-negative bacterium that is distributed widely throughout the world; it is mainly found in contaminated food, the poultry industry, and animal feces. The emergence of antibiotic-resistant *E. coli* poses a threat to human and animal health, which has led to renewed interest in phage-based therapy. *E. coli* biofilm control and prevention are of great importance. In this study, the isolated phages Flora and KM18 were found to belong to the family *Myoviridae*; the optimal preservation buffer was pH = 6~7, and the phage genome sizes were 168,909 (Flora) and 168,903 (KM18) bp. Phage Flora had a broader lytic spectrum than KM18. Phage Flora had a better antibiofilm effect than kanamycin sulfate in high-concentration *E. coli* cultures. A combination of the phage Flora and kanamycin sulfate showed better antibiofilm effects than Flora or kanamycin sulfate alone in low-concentration *E. coli* cultures. These characteristics can serve as a guideline for the selection of effective candidates for phage therapy, in this case antibiotic-resistant *E. coli* control in the poultry industry.

## Introduction

*Escherichia coli* is a gram-negative bacterium that is distributed widely worldwide; it is mainly found in contaminated food, the poultry industry, and animal feces, and some strains cause intestinal disease (1, 2). Moreover, *E. coli* O157:H7 is an important human pathogen causing abdominal pain, hemorrhagic colitis, gastroenteritis, diarrhea, and potentially fatal hemolytic-uraemic syndrome (HUS), hemorrhagic colitis, and death (3–5). The overuse of vancomycin, methicillin, trimethoprim, and sulfamethoxazole has led to the generation of many antibiotic-resistant *E. coli* strains (6–8). Furthermore, with the emergence of an increasing number of multidrug-resistant (MDR) bacteria, antibiotic substitutes are urgently needed (9, 10).

*E. coli* adhere and internalizes in epithelial cells. *E. coli* persistence in cattle mammary glands causes mastitis and biofilms formation related to recurrence (10). In addition, *E. coli* biofilms are related to antibiotic resistance and cattle mastitis. Bacterial biofilms are multicellular communities of microorganisms that embed within a self-produced extracellular matrix attached to non-biological, biological, and highly hydrated extracellular matrices on surfaces (11–13). The extracellular polymeric substances in the matrix of biofilms act as a barrier, reducing the penetration of antimicrobial agents into the interior of the biofilms (14, 15). Biofilms are highly resistant to desiccation, antibiotics, acidic conditions, and heat (16). Bacteria in biofilms are ~10–1,000 times less susceptible to antimicrobial agents than planktonic bacteria because the extracellular polymeric substances of the biofilm prevent contact with antimicrobial agents (17, 18). This makes the complete elimination of biofilms in animal husbandry, the food industry, and the clinic nearly impossible (19).

The abuse of antibiotics results in the problem of multiple resistant bacteria (MRB). In 2003, annually, 80,000 deaths were reported to be caused by the abuse of antibiotics in China (20). Phages and their derivatives are ideal candidates for replacing or compensating for antibiotic problems in the future (21). Phages have the ability to sterilize bacteria (22, 23). Phages appear to be a good alternative to antimicrobials and disinfectants for their ability to kill bacteria. Above all, phages only infect bacteria and are not harmful to humans, making them safe for application in the clinic and food products (24). A recent study found that phages have high efficiency in reducing and controlling bacterial biofilms on various surfaces, such as those produced by *Pseudomanas aeruginosa, Salmonella, E. coli*, and *Listeria monocytogenes* (25–28).

Gram-negative phages first attach to the surface receptor lipopolysaccharide via phage tail fiber proteins before they begin to infect bacteria (29). The narrow lytic spectrum restricts the application of phages. In this study, we isolated and characterized two lytic *E. coli* phages, flora and KM18. This study aimed to characterize the gene characteristics of the tail fiber of two different lytic spectrum phages. In addition, prevention and control of E. coli biofilm contamination are of great importance. The authors aimed to provide direction and a theoretical basis for the development and modification of broad-spectrum phages.

## Materials and Methods

### Bacterial Strains and Growth Conditions

*E. coli* strains were isolated from a hennery in Yunnan, China, and were used as host bacteria for phage isolation. The host strain and phage host range determination strains were grown aerobically on LB plates or in LB broth (Difco, Detroit, MI, USA) and incubated at 37°C. Soft top agar containing 0.5% (w/w) agar in LB broth was used for phage plaque confirmation, and LB agar plates containing 1.8% (w/w) agar were used for bacterial growth. All *E. coli* strains were stored at −80°C (Difco, Detroit, MI, USA) in 20% (v/v) glycerol.

### Phage Isolation and Purification

*E. coli*-targeting phages were isolated from a hennery and a foul water sewer. The phage isolation method was modified as follows (30). Briefly, 10 g of the hennery or foul water sewer sample was mixed with 30 ml of sterile normal saline (0.9% NaCl) buffered in a 50 ml sterile centrifuge tube and then shocked in an incubator at 180 rpm for 3 h at room temperature. Then, the samples were centrifuged at 4,500 × g for 10 min and filtered with a 0.22 μm filter membrane. A total of 15 ml of each filtered medium was added to 35 ml of LB broth containing 1% (v/v) of an *E. coli* overnight culture and then incubated for 2 d. After that, the cultures were centrifuged at 7, 000 × g for 10 min, and the supernatant was filtered with a 0.22 μm filter membrane. The filtrate was diluted by 10-fold serial dilutions, mixed with 6 ml of molten LB soft agar containing 200 μl *E. coli* (2 × 10^8^ cfu/ml), and immediately added to an LB plate. After overnight culture, plaque formation was observed. A single phage plaque was selected for phage purification, which was repeated three times.

### pH, Thermotolerance, MOI, and Growth Curve of the Isolated Phage

The phage flora stock was diluted to 1 × 10^8^ pfu/ml with LB broth. Liquid buffer (0.99 ml) with pH values of 3, 4, 5, 6, 7, 8, 9, 10, and 11 (50 mmol/L citrate buffer for pH 3, 4, and 5; 50 mmol/L phosphate buffer for pH 6, 7, and 8; 50 mmol/L Tris-HCl buffer for pH 9; 50 mmol/L sodium carbonate buffer for pH 10 and 11) was placed in a 2 ml sterile centrifuge tube, and 0.01 ml of diluted phage Flora with a titer of 1 × 10^6^ pfu/ml was added to each tube. The mixture was placed at room temperature for 1.5 h, and then the titer of phage Flora was assessed in different pH buffers. The experiments were repeated three times. Thermotolerance assessment was performed by placing 1.5 mL diluted phage Flora in temperature controllers at 4, 25, 37, 42, 50, 60, and 90°C for 1.5 h, respectively. The multiplicity of infection (MOI) is the ratio of phages to host bacteria used for the initial infection. Phage Flora stocks were added to the *E. coli* culture at MOIs of 0.0001, 0.001, 0.01, 0.1 1, 10, and 100, followed by incubation at 37°C for 10 h. The culture was centrifuged at 12, 000 × g for 15 min at 4°C, the supernatant was filtered with a 0.22 μm filter, and the titers of the phage Flora solutions were determined through the double plate method. The experiment was repeated three times. For growth curve measurements, 1 × 10^8^ pfu/ml phage Flora was added to an LB culture containing 1/250 of an *E. coli* seed culture based on the optimum MOI, and the culture was shaken at 37°C. Intermittent sampling was performed to determine the titer of phage Flora.

### Transmission Electron Microscopy

The morphology of the phage Flora particles was observed by transmission electron microscopy (TEM). Briefly, each phage stock dilution (~3 × 10^8^ to 3 × 10^9^ pfu/ml) was deposited on copper grids with carbon-coated Formvar films and stained with 2% uranyl acetate (pH 4.0). Phage Flora samples were imaged using a Philips EM 300 electron microscope operated at 80 kV at Ningbo University (Ningbo, China). Phage Flora was classified and identified according to the International Committee on Taxonomy of Viruses.

### Phage Genome DNA Extraction, Sequencing, and Bioinformatics Analysis

First, phage Flora was purified by concentrating on a high titer stock with a 10 kDa filter (~10^9^ to 10^10^ pfu/ml). Purified phage Flora was treated with DNase and RNase at 37°C for 1.5 h. Then, a Takara Minibest Viral RNA/DNA Extraction Kit (Cat# 9766) was used to obtain purified phage Flora genomic DNA. The restriction endonucleases *Eco*rI, *Not* I, *Hin*d III, and *Xho*l I were used for phage Flora genome digestion. Extracted phage Flora genomic DNA was sequenced using an Illumina HiSeq (Sangon Biotech, China). The original sequencing data were evaluated by FastQC and assembled with SPAdes assembler software. The NCBI Blast comparison with multiple databases of COG, KOG, CDD, NR, NT, PFAM, SwissProt, and TrEMBL were used for functional annotation information of the gene protein sequences.

### Phage Lytic Spectrum and Antimicrobial Susceptibility of *E. coli*

The host ranges of phages flora and KM18 were determined by the spot test method (31). The reference strains were tested for susceptibility to phages Flora and KM18. Generally, 250 μl reference strains (10^9^ cfu/ml) was added to 6 ml liquified LB soft agar (LB broth with 0.5% (w/w) agar) and poured over an LB 1.8% (w/w) agar plate. Four minutes later, single drops of the Flora and KM18 phage suspensions were added and incubated at 37 °C for 24 h. The antibiotic susceptibility of the *E. coli* strains was tested against 17 antibiotics by the minimal inhibitory concentration (MIC) method. The antimicrobials tested were penicillin, streptomycin, kanamycin sulfate, cefoxitin, ampicillin, ceftriaxone, gentamicin, ertapenem, aztreonam, amoxicillin, ciprofloxacin, imipenem, levofloxacin, cefepime, macrodantin, and amikacin.

### Structure and Molecular Diversity of *E. coli* Phage Tail Fibers

The amino acid sequences of the *E. coli* phages Flora and KM18 tail fibers were obtained by genomic sequencing. *E. coli* phage tail fiber tertiary structure homology modeling was performed using the SWISS-MODEL online suite. Phage tail fiber gene specificity was studied with BioEdit software.

### Assessment of the Effects of Phage Flora and Kanamycin Sulfate on Biofilm Formation

First, the phage preparations were made, and a 48-well-cell slide was placed into a 24-well-plate. The seed solution was inoculated into 100 ml of LB culture solution at a concentration of 4‰. About 1 ml of bacterial solution was inoculated into a 24-well-plate. In one group, phage Flora, kanamycin sulfate, or mixtures of kanamycin sulfate and phage Flora were added, with no addition used as a control (the phage was added at an MOI = 0.1; the final concentration of kanamycin sulfate was 10 μg/ml), followed by incubation (37 °C, 24 h). In the other group, *E. coli* were cultured first for 12 h, after which phage Flora, kanamycin sulfate, or mixtures of kanamycin sulfate and phage Flora were added, with no addition used as a control (the phage Flora was added at a MOI = 0.1; the final concentration of kanamycin sulfate was 10 μg/ml), followed by incubation (37°C, 12 h). The cfu count of each sample was measured through the plate counting method. Next, the recovered culture was washed twice with PBS buffer and fixed with 2.5% pre-cooled glutaraldehyde at room temperature for 2 h in the dark. The samples were washed twice with PBS buffer and then dehydrated with an increasing ethyl alcohol gradient (15, 30, 40, 50, 70, 100% v/v) for 15 min for each step. Afterward, the samples were dried overnight and gilt and the results were obtained through scanning electron microscopy with an accelerating voltage of 20 kV. The *E. coli* seed solution was inoculated in LB at a concentration of 4‰ for overnight culture. Then, a 200 times dilution with LB was performed in the 96-well-plate (200 μl/well), and each sample had three replicate wells. For one group, phage Flora, kanamycin sulfate, or mixtures of kanamycin sulfate and phage Flora were added, with no addition used as a control (the phage Flora was added at an MOI = 0.1; the final concentration of kanamycin sulfate was 10 μg/ml), followed by incubation (37 °C, 24 h). In the other group, *E. coli* was first cultured for 12 h, after which phage Flora, kanamycin sulfate, or mixtures of kanamycin sulfate and phage Flora were added, with no addition used as a control (the phage Flora was added at an MOI = 0.1; the final concentration of kanamycin sulfate was 10 μg/ml), followed by incubation (37°C, 12 h). The *E. coli* population density (OD_600_) was measured using an ELISA (Thermo Scientific, EUA), and the bacterial solution was discarded. Each well was washed twice with PBS to remove unattached *E. coli*, which was repeated three times. Then, 99% methanol was added, and the cells were fixed for 10 min. Then, the methanol was discarded, and the well was dried at room temperature, followed by the addition of 2% crystal violet for 10 min. The culture plate was rinsed with running water until the water was colorless. After drying, the absorbance was measured at a wavelength of 570 nm with a microplate reader. The experiment was repeated three times.

## Results

### Characteristics and Morphology of the Isolated Phages

**Figure 1:**
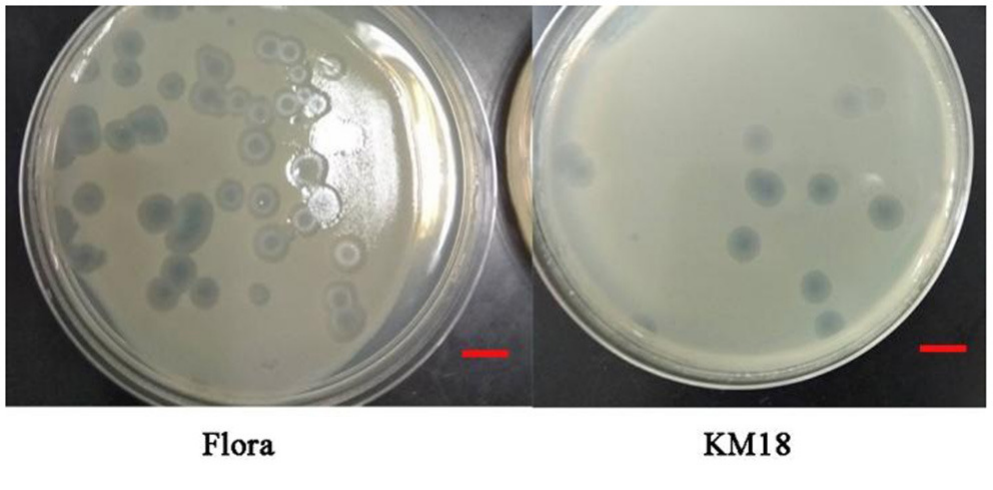
Plaques formed by *E. coli* phages KM18 and Flora; the host strain was *E. coli* BL21 incubated overnight at 37°C. The scale was 1 cm.

**Figure 2:**
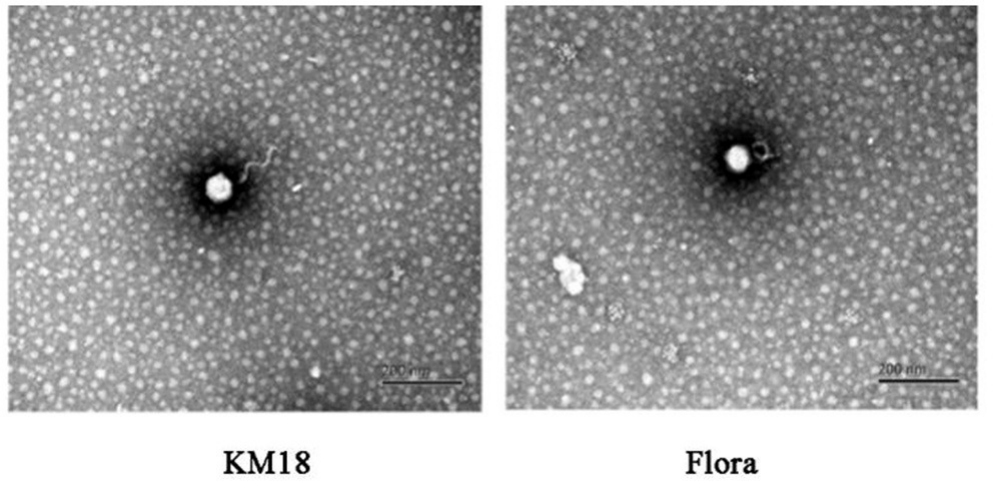
Morphological features of *E. coli* phages KM18 and Flora as demonstrated by transmission electron microscopy (TEM).

**Figure 3:**
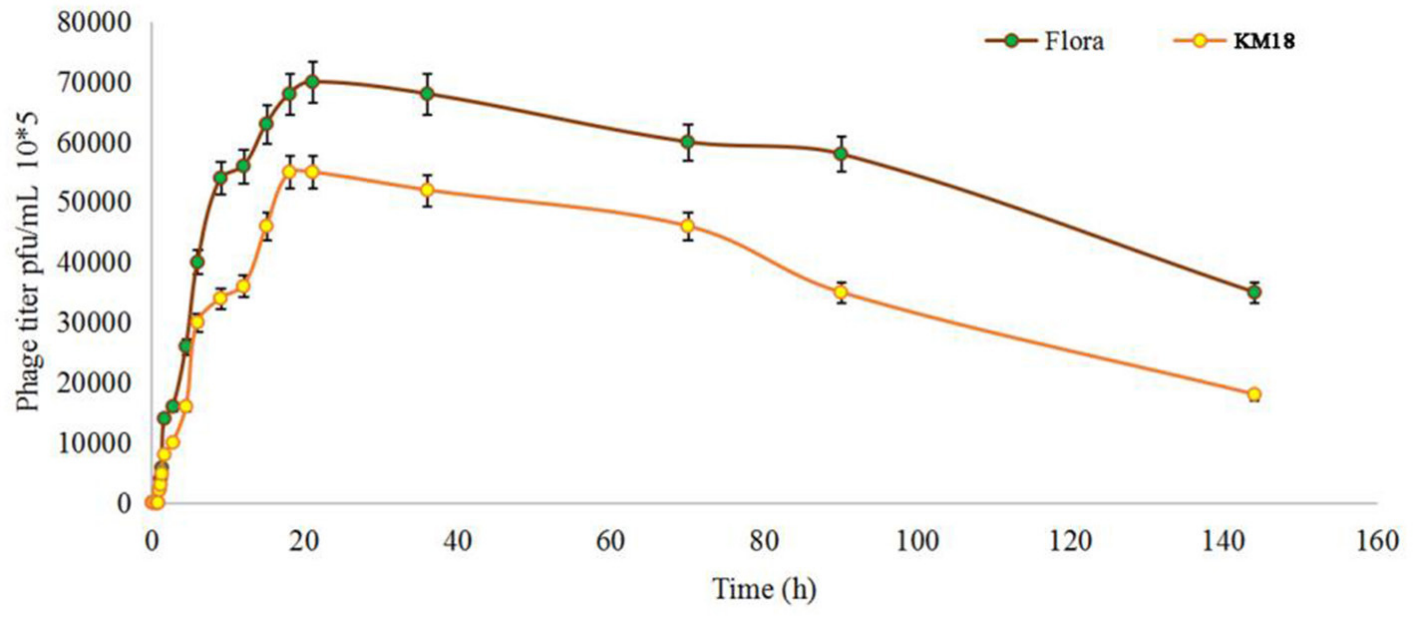
Population dynamics of phages KM18 and Flora incubated with *E. coli* BL21.

Virulent *E. coli* phage Flora and KM18 were isolated from a hennery and a foul water sewer in Yunnan, China. The plaques of phages Flora and KM18 appeared 6–8 and 5–7 mm in diameter, respectively, after overnight incubation at 37°C (Figure 1). Negative staining of purified *E. coli* phages Flora and KM18 was observed using an electron microscope. TEM revealed the phages Flora and KM18 virions to have an icosahedral head of 60 ± 2 nm in diameter and a contractile tail of 150 ± 5 nm in length (Figure 2). The morphology of phages Flora and KM18 indicated that they belong to the family *Myoviridae*. A one-step growth curve of the phages KM18 and Flora was obtained by inoculation of *E. coli* at an MOI of 1 at 37 °C (Figure 3). The latent period for the phages KM18 and Flora was 55 min. The titers of phages KM18 and Flora reached peaks very quickly in 20 h and appeared to decrease after 20 h. The burst size of phages KM18 and Flora was ~2000.

### Optimum Temperature, pH, and MOI of the Isolated Phages

**Figure 4:**
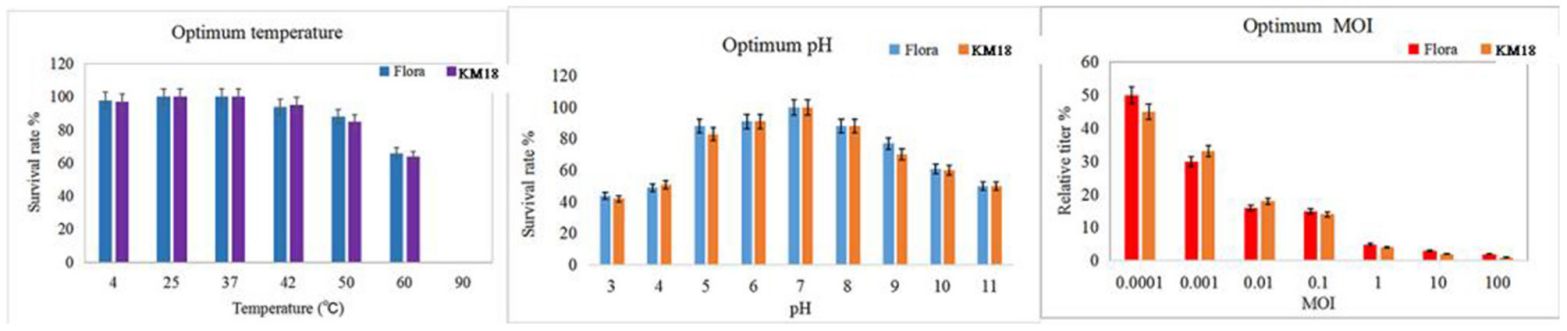
Optimum temperature, pH, and MOI of the isolated phages KM18 and Flora.

Phages KM18 and Flora had the highest viability after treatment for 1 h at 37°C, with a noticeable decline at 60 °C and complete inactivation at 90 °C (Figure 4). The results showed that phages KM18 and Flora were viable at low temperatures, which is consistent with the optimum survival temperature of *E. coli*. Phages KM18 and Flora showed the most plaques at pH = 7; furthermore, phages KM18 and Flora had a high activity at pH = 11 and pH = 3 (Figure 4). These results indicated that phages KM18 and Flora have good tolerance to alkali and acids. MOI refers to the ratio of the number of phages to bacterial cells. The optimum MOI of phages KM18 and Flora was 0.0001; among the MOIs tested, plaques of Flora and KM18 decreased significantly starting at MOI = 0.0001 and reaching a minimum at MOI = 100 (Figure 4).

### Characterization and Analysis of the Phage Genomes

**Figure 5:**
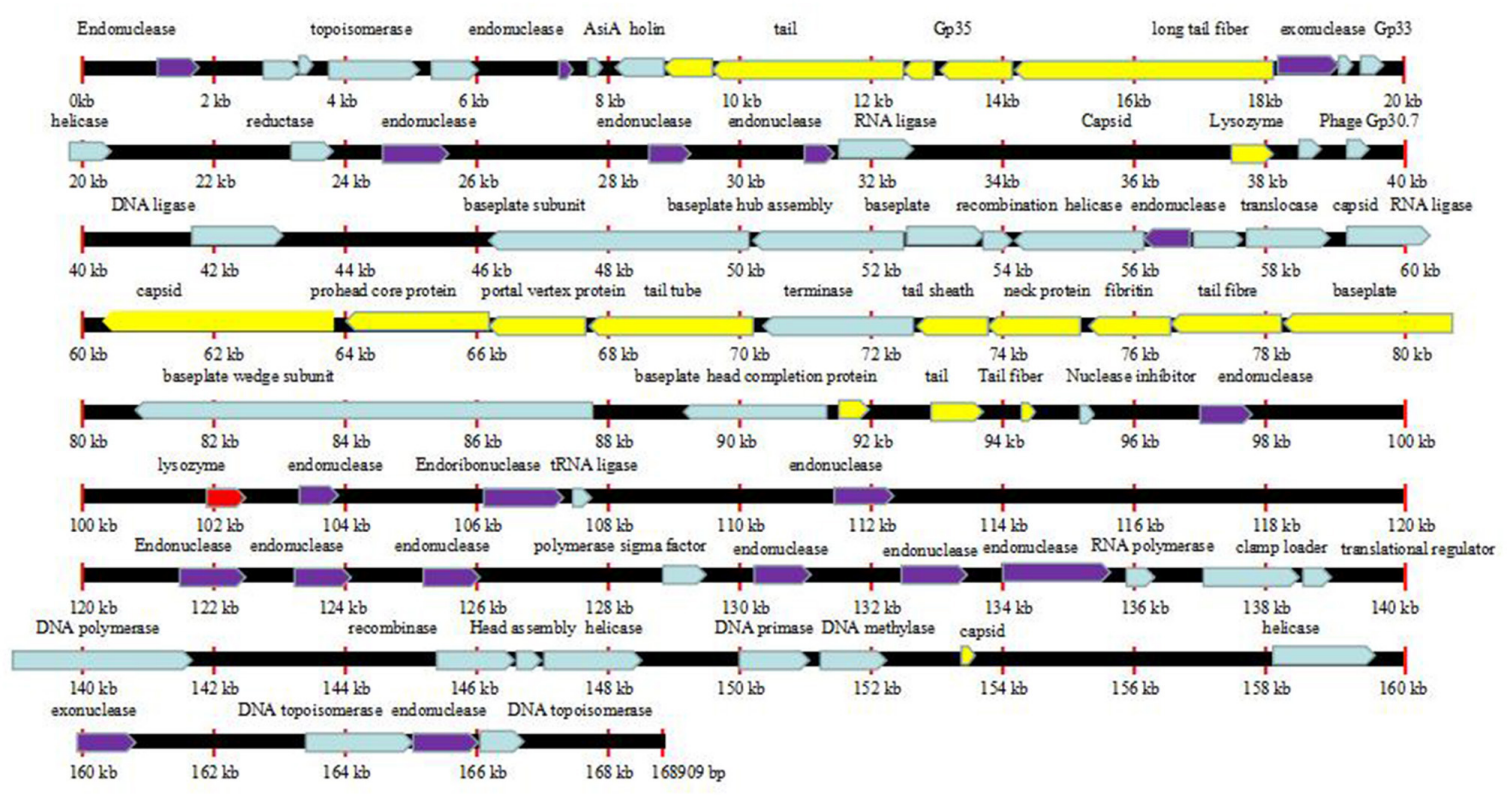
Line map of the phage Flora genome. In the Flora map, genes colored red represent lysozymes, genes colored purple represent endonucleases, and genes colored yellow represent tail structure genes. The arrows represent the ORFs and indicate the direction of transcription.

The complete genome sizes of phages Flora and KM18 were 161,903 and 161,909 bp, respectively. We identified 263 protein-coding genes [open reading frames (ORFs)] in Flora. Genome analysis revealed that phages Flora and KM18 are virulent phages (Figure 5). The complete genomic sequences of phage Flora and KM18 were deposited into the NCBI GenBank database (https://www.ncbi.nlm.nih.gov/nuccore) (MT787017 and MT787018).

### Phage Lytic Spectrum and Antibiotic Resistance of *E. coli*

*E. coli* strains were isolated from a hennery in Yunnan, China. Unfortunately, they showed a broad spectrum of resistance (Table 1), but fortunately, some of them could be removed by phages Flora and KM18 (Table 2). All the *E. coli* strains possessed resistance to penicillin, streptomycin, kanamycin sulfate, ertapenem, amoxicillin, imipenem, cefepime, macrodantin, and amikacin but were susceptible to ampicillin and ceftriaxone. Lytic *E. coli* phage Flora was able to infect 4/10 *E. coli* strains that were isolated from the hennery in Yunnan, China (Table 2). This analysis demonstrated the wide host range of the isolated phage Flora.

**Table 1:** Antibiotic resistance of *Escherichia coli* isolates used in this study.

	***Escherichia coli***											
Antibiotic	A	B	C	D	E	F	G	H	I	J	DH5α	BL21
Penicillin	S	S	S	S	S	S	S	S	S	S	S	S
Streptomycin	S	S	S	S	S	S	S	S	S	S	S	S
kanamycin sulfate	S	S	S	S	S	S	S	S	S	S	S	S
Cefoxitin	S	S	S	S	S	S	S	S	S	S	S	S
Ampicillin	R	R	R	R	R	R	R	R	R	R	R	R
gentamicin	R	S	R	R	S	S	S	S	S	R	S	S
Aztreonam	R	S	R	S	S	R	R	S	S	S	S	R
Ciprofloxacin	R	R	R	R	S	R	R	R	S	S	S	S
Levofloxacin	R	R	R	S	S	R	R	S	S	S	S	S
Macrodantin	S	S	S	S	S	S	S	S	S	S	S	S
Amikacin	S	S	S	S	S	S	S	S	S	S	S	S
Cefepime	S	S	R	R	S	R	S	S	S	S	S	S
Imipenem	S	S	S	S	S	S	S	S	S	S	S	S
Amoxicillin	R	S	R	S	S	R	S	S	S	R	S	S
Ertapenem	S	S	S	S	S	S	S	S	S	S	S	S
Ceftriaxone	R	S	R	R	R	R	R	R	S	R	R	R

*S, susceptible; R, resistant*.

**Table 2:** Host range analysis of phages KM18 and Flora.

**Strain**	**KM18**	**Flora**
*Escherichia coli*-A	**+**	**+**
*Escherichia coli*-B	**-**	**+**
*Escherichia coli*-C	**-**	**-**
*Escherichia coli*-D	**-**	**-**
*Escherichia coli*-E	**-**	**+**
*Escherichia coli*-F	**-**	**+**
*Escherichia coli*-G	**-**	**-**
*Escherichia coli*-H	**-**	**-**
*Escherichia coli*-I	**-**	**-**
*Escherichia coli*-J	**+**	**-**
*Escherichia coli*-DH5α	**-**	**+**
*Escherichia coli-*BL21	**+**	**+**

*clear lysis zone **(+)**, no lysis zone **(−)***.

### Structure and Molecular Diversity of the *E. coli* Phage Tail Fibers

**Figure 6:**
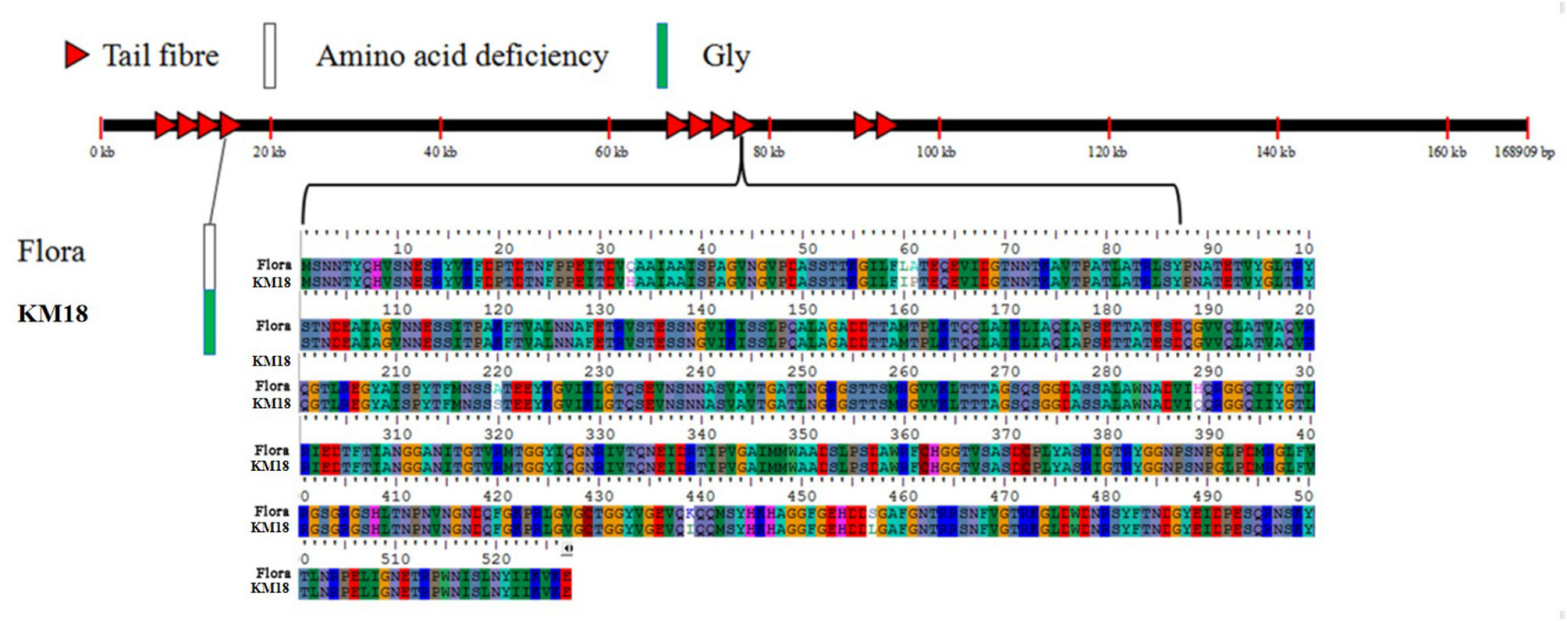
Location of mutated bases and amino acids in the *E. coli* phage tail fiber proteins of the Flora and KM18 genomes. There are eight base mutations in the whole genome tail fiber; these positions are 322(-/G) of the fourth tail fiber and 33(G/A), 60(A/G), 61(A/G), 220(A/G), 289(A/G), 439(A/G), and 457(A/G) of the eighth tail fiber.

**Figure 7:**
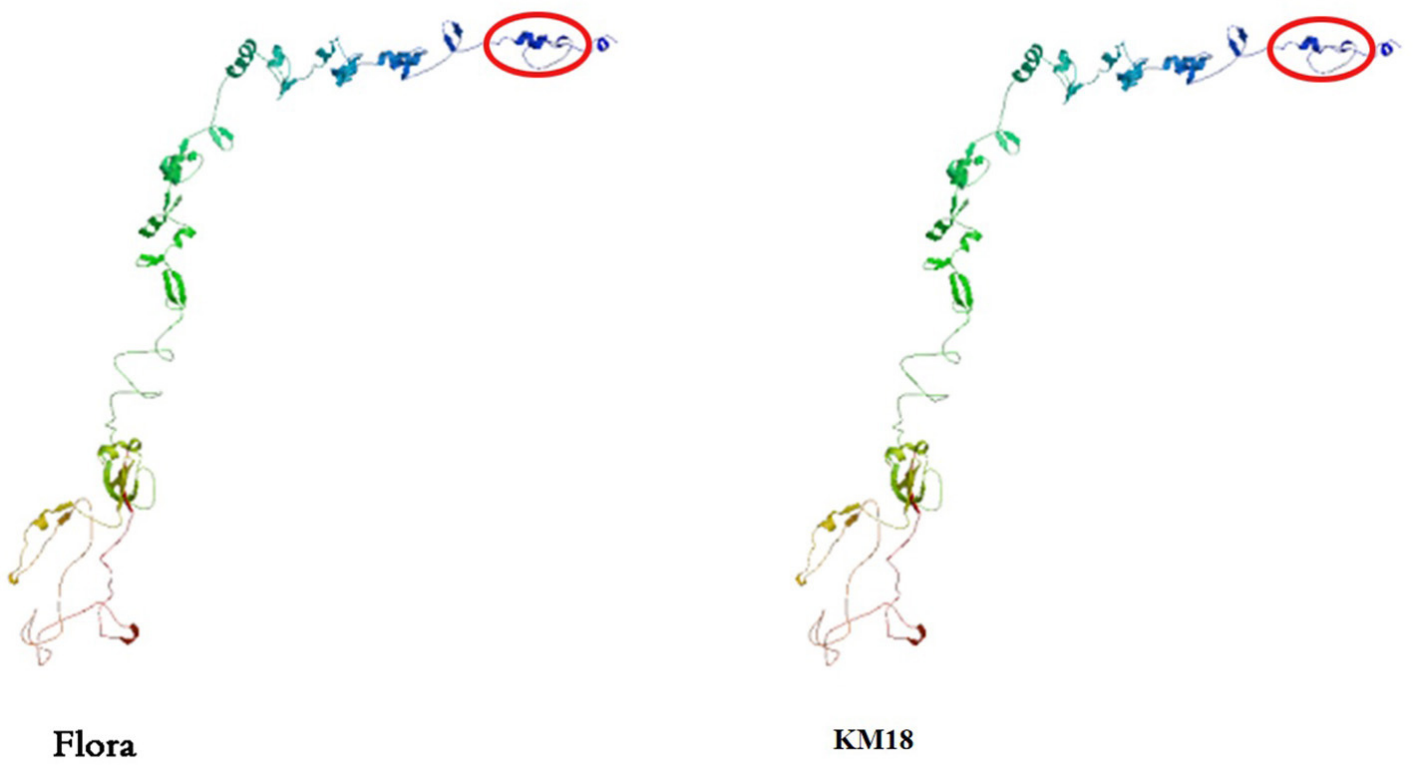
Predicted tertiary structure of the *E. coli* phage eighth tail fiber protein. Compared with the KM18 eighth tail fiber, Flora has special β strands in the eighth tail fiber.

Tail fiber adsorption to bacteria is the first step in phage infection. The *E. coli* phage tail fiber sequences and sizes have significantly different numbers of amino acids and homologies. Genome sequencing revealed that Flora has 10 different tail fiber genes of 8711-9262, 9290-12370, 12374-13044, 14234-18100, 67787-68278, 68395-70374, 72725-73543, 76778-78361, 92957-93487, and 94262-94504 bp in its genome. The identity and homology of the *E. coli* phage tail fiber genes were analyzed through BioEdit software. Amino acid differences between Flora and KM18 were located at positions 33 (Q-H), 60 (L-I), 61 (A-P), 220 (A-S), 289 (H-Q), 439 (K-I), and 457 (S-L) of 76,778-78,361 bp in the genome. Compared with the KM18 genome, the Flora Gly amino acid deletion was located at position 322 of 14,234-18,100 bp in the genome (Figure 6). The *E. coli* phage tail fiber tertiary structure was predicted by SWISS-MODEL (https://swissmodel.expasy.org/). The tail fiber tertiary structures were highly similar between Flora and KM18 and had the same modeling template (https://swissmodel.expasy.org/templates/5iv5.15.A). Compared with KM18 tail fiber homologous modeling, Flora has special β-strands (Figure 7).

### Comparison of the Effects of Phage Flora and Kanamycin Sulfate on Host Biofilm Formation

**Figure 8:**
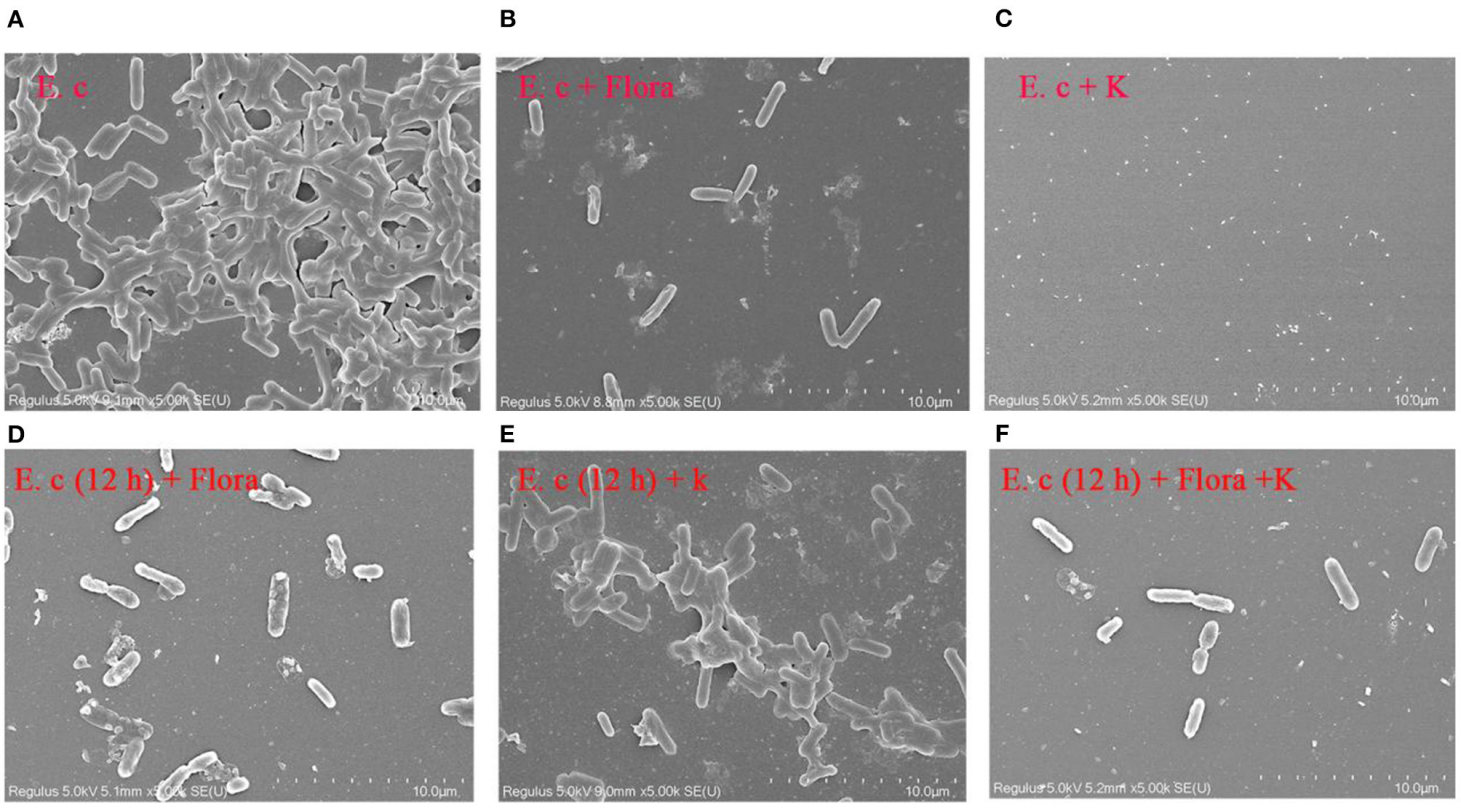
Scanning electron micrographs (SEM) of *E. coli* colonization before and after phage Flora (MOI = 1) and kanamycin sulfate (10 μg/ml) application to the biofilms formed on round coverslips. **(A)** *E. coli* was inoculated at a concentration of 4‰ and cultured for 24 h, followed by **(B)** addition of phage Flora (MOI = 1) or **(C)** addition of kanamycin sulfate (10 μg/ml). **(D)** *E. coli* was inoculated at a concentration of 4‰ and cultured for 12 h, after which phage Flora (MOI=1) was added and cultured for 12 h. **(E)** *E. coli* was inoculated at a concentration of 4‰ and cultured for 12 h, after which kanamycin sulfate (10 μg/ml) was added and cultured for 12 h. **(F)** *E. coli* was inoculated at a concentration of 4‰ and cultured for 12 h, after which phage Flora (MOI = 1) and kanamycin sulfate (10 μg/ml) was added and cultured for 12 h (5, 000× magnification).

**Figure 9:**
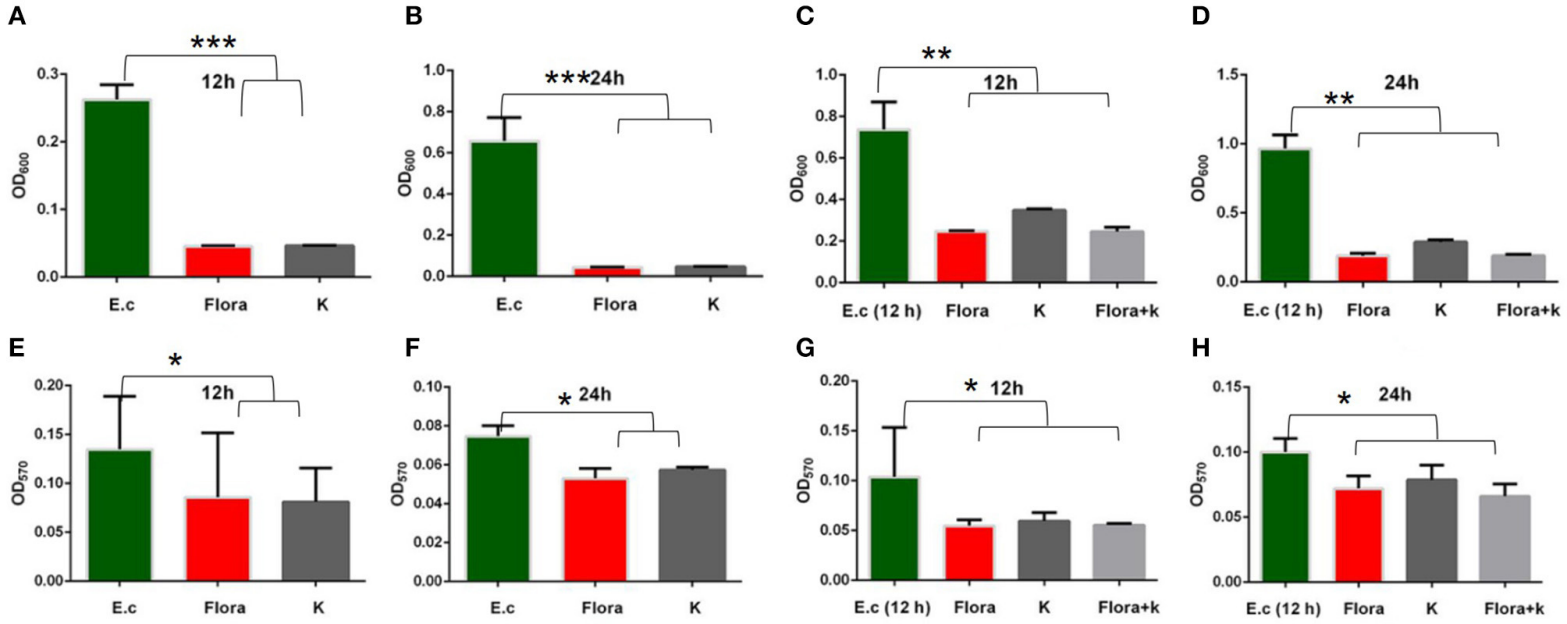
Effects of phages and kanamycin sulfate (10 μg/ml) on biofilms. **(A,B)** Effects of phage Flora and kanamycin sulfate (10 μg/ml) on *E. coli* (inoculated at a concentration of 4‰) growth after 12 and 24 h of culture (OD_600_). **(E,F)** Effects of phage Flora and kanamycin sulfate (10 μg/ml) on *E. coli* (inoculated at a concentration of 4‰) biofilms cultured for 12 and 24 h (OD_570_). **(C,D)** Effects of phage Flora and kanamycin sulfate (10 μg/ml) on *E. coli* (inoculated at a concentration of 4‰) growth after first being cultured for 12 h, followed by phage Flora and kanamycin sulfate (10 μg/ml) addition and culturing for 12 and 24 h (OD_600_). **(G,H)** Effects of phage Flora and kanamycin sulfate (10 μg/ml) on *E. coli* (inoculated at a concentration of 4‰) biofilms after first being cultured for 12 h, followed by phage Flora and kanamycin sulfate (10 μg/ml) addition and culturing for 12 and 24 h (OD_570_). *p < 0.05, **p < 0.01, ***p < 0.001.

**Figure 10:**
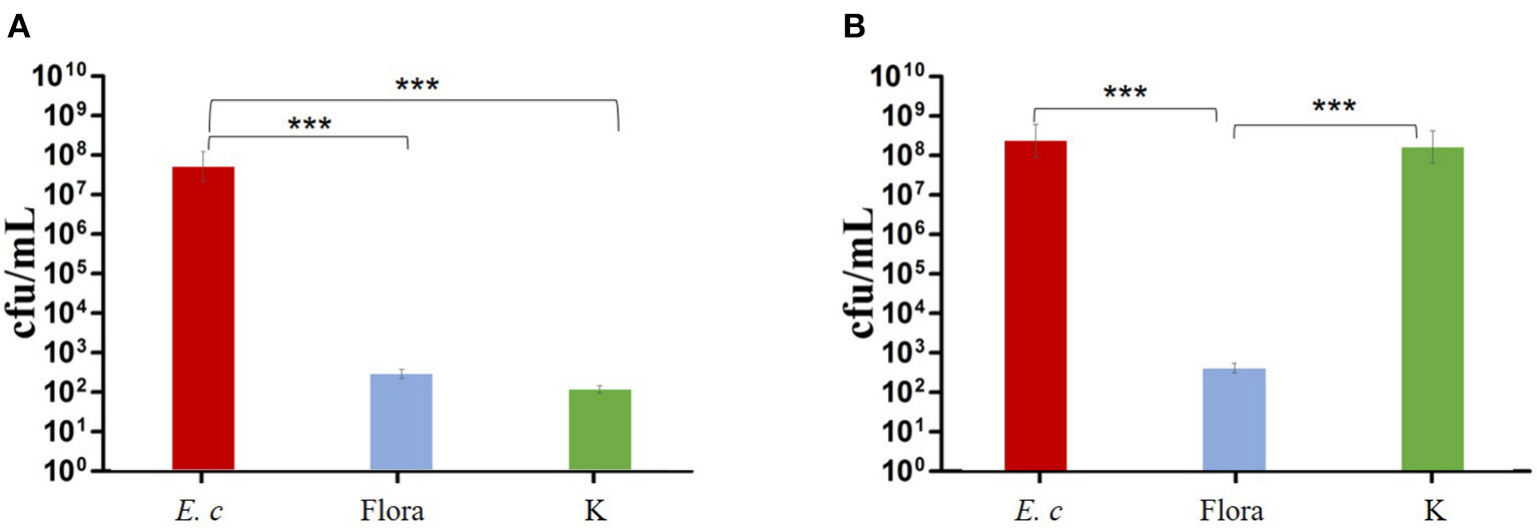
Effects of phage and kanamycin sulfate (10 μg/ml) on *E. coli* colony-forming units. **(A)** Effects of phage Flora and kanamycin sulfate (10 μg/ml) on *E. coli* (inoculated at a concentration of 4‰) cultured for 24 h. **(B)** Effects of phage Flora and kanamycin sulfate (10 μg/ml) on *E. coli* (inoculated at rate of 4‰) after first being cultured for 12 h, followed by phage Flora and kanamycin sulfate (10 μg/ml) addition and culturing for 12 h. ***p < 0.001.

SEM was used to assess *E. coli* biofilm formation on round coverslips treated with phage Flora (MOI = 0.1) and kanamycin sulfate (10 μg/ml). After *E. coli* inoculation at a concentration of 4‰, phage Flora (MOI = 0.1) and kanamycin sulfate (10 μg/ml) were added immediately and cultured for 24 h. Kanamycin sulfate showed a better eradication effect than phage Flora according to the results of the microplate reader OD_570_ of the *E. coli* biofilm, the scanning electron micrograph, and the OD_600_ of the bacterial culture solution (Figures 8–**10**). Nevertheless, when *E. coli* was inoculated at a concentration of 4‰ and cultured for 12 h, followed by the addition of phage Flora (MOI = 0.1) and kanamycin sulfate (10 μg/ml) and culturing for 12 h, phage Flora showed a better eradication effect than kanamycin sulfate according to the results of the microplate reader OD_570_ of the *E. coli* biofilm, the scanning electron micrograph and the OD_600_ of the bacterial culture solution (Figures 8–**10**). In addition, when *E. coli* was inoculated at a concentration of 4‰ and cultured for 12 h, followed by phage Flora (MOI = 0.1) and kanamycin sulfate (10 μg/ml) addition and culturing for 12 h, the combination of phage Flora and kanamycin sulfate showed a better eradication effect than phage Flora or kanamycin sulfate alone according to the results of the microplate reader OD_570_ of the *E. coli* biofilm, the scanning electron micrograph and the OD_600_ of the bacterial culture solution (Figures 8–**10**). The results of the *E. coli* colony-forming units indicated that the combination of phage Flora and kanamycin sulfate showed a better eradication effect than phage Flora or kanamycin sulfate alone (**Figure 10**).

## Discussion

The *E. coli* strains used in this study were isolated from a hennery in Yunnan, China; unfortunately, they were resistant to penicillin, streptomycin, kanamycin sulfate, ertapenem, amoxicillin, imipenem, cefepime, macrodantin, and amikacin. The emergence of MDR strains urgently requires new measures to inhibit pathogens. With the overuse of antibiotics, multi-drug-resistant bacteria and superbacteria have led to public health problems (1, 7, 8, 32). The isolated *E. coli* phage Flora was identified as an ideal substitute for antibiotics due to its strong lytic activity and wide lytic spectrum. Moreover, phage Flora showed a better eradication effect than kanamycin sulfate in a high-concentration culture, and the combination of phage Flora and kanamycin sulfate showed a better eradication effect than phage Flora or kanamycin sulfate alone in a low-concentration culture (Figures 8–**10**) (33).

The isolated *E. coli* phages Flora and KM18 belong to the family *Myoviridae*, and the genome sizes were 168,909 and 168,903 bp, respectively. In comparison, the *E. coli* phage phiLLS genome size is 107,263 bp, and the genome sizes of phage vB_EcoM-Ro111lw, vB_EcoS-Ro145lw, and vB_EcoM-Ro157lw are between 42 kb and 149 kb; moreover, they all belong to the family *Myoviridae* (32). Washizaki et al. constructed a phage T4 mutant strain Arl and identified that the amino acid changes at V941E, A955E, and G943S in the DT region of the phage tail fiber played an important role in the interaction with lipopolysaccharide, indicating that the terminal head domain of T4 tail fiber was interacting with lipopolysaccharide (34). The genome characteristics of *E. coli* phages Flora and KM18 revealed that Flora encodes a special endonuclease and a more unique exonuclease. The extra endonuclease and exonuclease in Flora may be the reason why it has a wider lytic spectrum. Another reason for the wider lytic spectrum of Flora could be its greater resistance than KM18 to restriction enzymes.

The ability to form biofilms on different food surfaces increases the risk of microorganism cross-contamination, particularly in poultry products, which is a serious problem for food industries, clinics, and public health (35–37). However, the significant problem of pathogen biofilm elimination is still challenging (38). Until now, there has been no ideal technology for biofilm control; hence, new control strategies for biofilms are constantly recommended (39). In this study, we demonstrated that phage Flora has better properties than antibiotics for reducing the biofilm formation of *E. coli* (Figures 8, 9).

The results showed that phage Flora and kanamycin sulfate have the ability to reduce *E. coli* biofilms. Kanamycin sulfate showed a better antibiofilm effect than phage Flora in low-concentration *E. coli* cultures (Figures 8–10). Nonetheless, phage Flora showed a better antibiofilm effect than kanamycin sulfate in high-concentration *E. coli* cultures (Figures 8–10). The data of this study provide strong evidence that the application of phage Flora could reduce the growth of *E. coli* biofilms, which is important for maintaining public health.

In conclusion, we first isolated and characterized lytic *E. coli* phages and found that the combination of phage and antibiotics shows significantly better antibiofilm and bactericidal properties than either antibiotics or phages alone. The data of this study provide strong evidence that phage application could reduce *E. coli* biofilm growth, which is important for maintaining public health.

## Data Availability Statement

The original contributions presented in the study are included in the article/supplementary material, further inquiries can be directed to the corresponding author.

## Author Contributions

CL and RZ conceived and designed the experiments. LJ performed the experiments, analyzed the data, and wrote the paper. WL, YJ, and LJ contributed reagents, materials, and analysis tools. All authors read and approved the final manuscript.

## Funding

This work was supported by the General project of Education Department of Zhejiang Province (Y202043742), the Research Fund Program of Guangdong Provincial Key Lab of Pathogenic Biology and Epidemiology for Aquatic Economic Animals (No. PBEA2020ZD01), the Natural Science Foundation of Ningbo (2021J118 and 2021J113), the National Natural Science Foundation of China (82160403), and the K.C. Wong Magna Fund at Ningbo University.

## Conflict of Interest

The authors declare that the research was conducted in the absence of any commercial or financial relationships that could be construed as a potential conflict of interest.

## Publisher's Note

All claims expressed in this article are solely those of the authors and do not necessarily represent those of their affiliated organizations, or those of the publisher, the editors and the reviewers. Any product that may be evaluated in this article, or claim that may be made by its manufacturer, is not guaranteed or endorsed by the publisher.
